# Helicobacter pylori infection: approach of primary care physicians in a developing country

**DOI:** 10.1186/1471-230X-9-23

**Published:** 2009-04-09

**Authors:** Shahid Ahmed, Mohammad Salih, Wasim Jafri, Hasnain Ali Shah, Saeed Hamid

**Affiliations:** 1 Section of Gastroenterology, Department of Medicine, Aga Khan University Hospital, Karachi, Pakistan

## Abstract

**Background:**

The aim of the study was to assess the knowledge and practices of primary care physicians in diagnosis and management of *Helicobacter pylori (H. pylori) *infection in developing country.

**Methods:**

This convenient sample based, cross sectional study was conducted in primary care physicians of Karachi, Pakistan from March 2008 to August 2008 through a pretested self-designed questionnaire, which contained 11 items pertaining to *H. pylori *route of transmission, diagnosis, indication for testing, treatment options, follow up and source of information.

**Results:**

Out of 509 primary care physicians, 451 consented to participate with the response rate of 88.6%. Responses of 426 primary care physicians were analyzed after excluding 19 physicians. 78% of the physicians thought that contaminated water was the source of spread of infection, dyspepsia was the most frequent indication for investigating *H. pylori *infection (67% of the physicians), while 43% physicians were of the view that serology was the most appropriate test to diagnose active *H. pylori *infection. 77% of physicians thought that gastric ulcer was the most compelling indication for treatment, 61% physicians preferred Clarithromycin based triple therapy for 7–14 days. 57% of the physicians would confirm *H. pylori *eradication after treatment in selected patients and 47% physicians preferred serological testing for follow-up. In case of treatment failure, only 36% of the physicians were in favor of gastroenterologist referral.

**Conclusion:**

The primary care physicians in this study lacked in knowledge regarding management of *H. pylori *infection. Internationally published guidelines and World gastroenterology organization (WGO) practice guideline on *H. pylori *for developing countries have little impact on current practices of primary care physicians. We recommend more teaching programs, continuous medical education activities regarding *H. pylori *infection.

## Background

The world wide prevalence of *H. pylori *is more than 50% [1,2]. It is more prevalent in developing countries as compared to developed countries [3]. Its prevalence in South Asia is ranging between 55 to 90% [4].

*H. pylori *infection is prevalent in more than 90% of peptic ulcer disease patients and the risk of bleeding from peptic ulcer increases by 1.79 times in patients with this infection as compared to controls [5]. Its successful eradication can prevent the ulcer relapse [6-8]. Similarly, in patients with non-ulcer dyspepsia the eradication of *H. pylori *may lead to improvement of symptoms [9].

In 1983, the concepts of etiology, pathogenesis and management of upper gastrointestinal (UGI) diseases have changed altogether after the successful isolation of *H. pylori *organism [10]. This has led to a number of *H. pylori *related information and the development of international guidelines. Majority of patients with dyspepsia are managed by primary care physicians and several educational initiatives have been undertaken to educate them regarding the appropriate diagnosis and management of this infection [11,12]. However, the results from several internationally published surveys from different developed countries have revealed that significant confusion still exists and discrepancies are present in the understanding of *H. pylori *among primary care physicians with respect to the pathogenesis, diagnosis and treatment [10]. The major uncertainties are in the management of patients with dyspepsia where the primary care physicians need to make a decision whether to test for *H. pylori *infection and treat if positive, or refer patients to a specialist [13,14].

There is scarce data from South Asia regarding the knowledge and practices of primary health care providers about *H. pylori *infection. The aim of this study was to assess the knowledge and practices of primary care physicians in the diagnosis and management of *H. pylori *infection in our country and to highlight the gaps in knowledge and management so that these areas can be focused in future continuous medical educational activities for primary care physicians.

## Methods

### Study design

This was cross sectional study in primary care physicians of Karachi, Pakistan. In this survey non probability convenient sampling technique was used.

### Sample size

We calculated sample size based two facts; the number of primary care physician in the city (4025, physicians) http://www.paksehat.com/[15] and the assumption that 50% of the practicing physicians lack in proper knowledge regarding management of *H. pylori *infection. With 95% confidence level, bound on error of 5% a sample size of 352 was calculated. The sample size was further inflated by 20% to adjust for non-responders, so the final sample size of 422 participants was calculated.

### Sample selection

#### Inclusion criteria

• Practicing primary care physicians seeing more than 20 patients per day in clinic and in practice for more than 2 years.

#### Exclusion Criteria

Primary care physicians were excluded if they fulfilled any of the following criteria;

• Non practicing primary care physicians,

• Physicians who have postgraduate diploma in medicine,

• Physicians who have attended national or international conferences on *H. pylori*/peptic ulcer disease management or related seminar/work shop in past 2 years.

## Data collection procedure

This cross sectional study was conducted from March 2008 to August 2008. A self administered pretested proforma with closed ended questions was used (see Additional file 1). The questionnaire contained 11 items pertaining to *H. pylori *infections; the route of transmission, knowledge of diagnosis, indication for testing, treatment options, duration of treatment, treatment regimens, follow up testing, referral to specialist and source of information of the primary care physicians. After written informed consent, the responses of primary care physicians were recorded on questionnaire during various non-related CME activities organized by the Aga Khan University Hospital and also the physicians were approached in their respective clinics on convenient basis. A total of 509 primary care physicians were approached with the response rate of 88.6%. Proforma was filled individually by each primary care physician without the help of any other person.

This study was also approved by Ethical review committee of The Aga Khan University Hospital, Karachi, Pakistan.

## Data analysis procedure

Statistical Packages for Social Sciences (S.P.S.S.) version 16 (Chicago Inc.) was used for entering and analysis of data. Charts and graphs were plotted using computerized program Microsoft Office (XP Professional) 2003.

The results were expressed as mean ± standard deviation or median with ranges for all continuous variables (e.g., age, years of practice, antibiotic use in days, PPI use in days) and numbers (percentage) for categorical data (e.g., gender, source of information about *H. pylori*).

## Results

Out of 509 primary care physicians, 451 consented to participate with the response rate of 88.6%. 19 were excluded as they had attended the workshops or seminars on *H. pylori *infection in last 2 years and proforma of 6 physicians were incomplete, so finally the responses of 426 primary care physicians were analyzed.

### Socio-demographic characteristics

The mean age and working years of the primary health care physicians were 42.38 ± 9.31 and 13.38 ± 7.73 years respectively; 315(73.94%) of primary health care physicians were males.

The responses of the primary care physicians to different questions (Study questionnaire attached) related to *H. pylori *infection are as follows:

#### 1) Route of transmission for H. pylori infection

**Figure 1:**
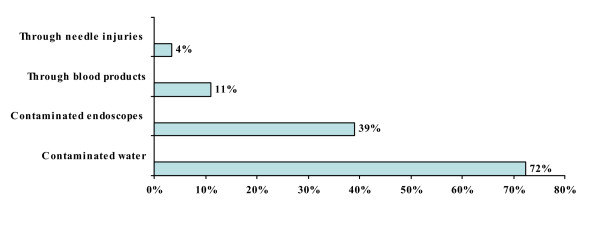
**Route of transmission for *H. pylori *infection**.

The knowledge about the route of transmission was checked through options like, contaminated water, contaminated endoscopes, blood products or needle injures. They could select more than one option. Out of 426 primary care physicians, 308 (72.3%) thought that contaminated water is mainly the source of spread, (Figure 1).

#### 2) Indications for testing H. pylori infection

**Figure 2:**
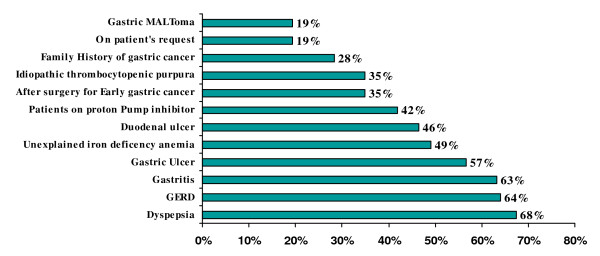
**Indications for testing *H. pylori *infection**.

Out of 426 primary care physicians, 287 (67%) opted for dyspepsia as the number one indication for investigating *H. pylori*, followed by gastroesophageal reflux diseases (GERD), gastritis, gastric and duodenal ulcers, iron deficiency anemia. Details of responses are given in Figure 2. Physicians could select any number of indications.

#### 3) Appropriate test for detection of active H. pylori infection

**Figure 3:**
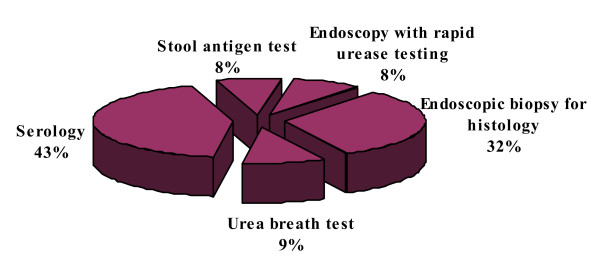
**Appropriate test for detection of active *H. pylori *infection**.

The physicians were asked which one diagnostic test they would choose for active *H. pylori *infection. As the single best test; 183 (43%) physicians would check serology (IgG antibody) for diagnosis (Figure 3).

#### 4) Treatment indications for H. pylori infection

**Figure 4:**
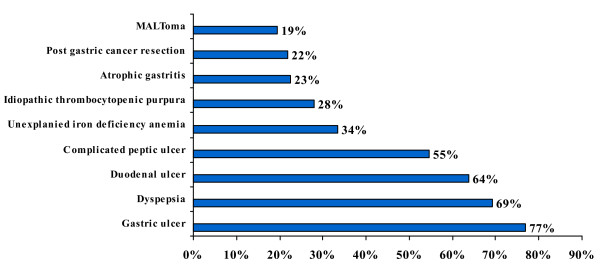
**Treatment indications for *H. pylori *infection**.

Physicians were asked when they would treat *H. pylori*. They were given with various options, shown in figure 4 and were allowed to select as many as they thought were appropriate. In reply 328 (77%) primary care physicians thought that gastric ulcer is the most compelling indication for the *H. pylori *eradication (Figure 4).

#### 5) H. pylori eradication in non ulcer dyspepsia

**Figure 5:**
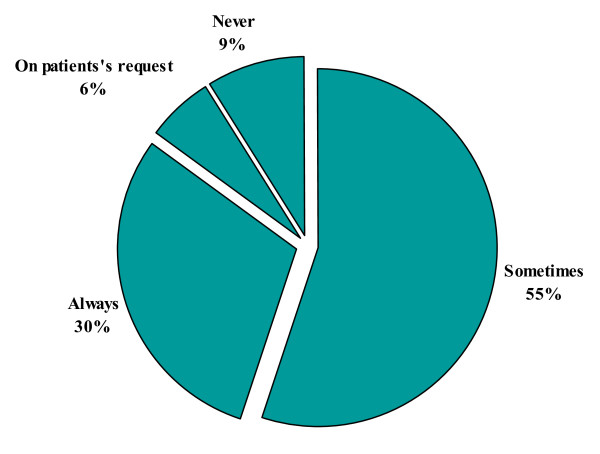
***H. pylori *eradication in non ulcer dyspepsia**.

Physicians were asked whether *H. pylori *eradication in non ulcer dyspepsia (NUD) was always/sometimes/on the patient request or never required. On one best selection basis 237 (55%) physicians would sometimes eradicate *H. pylori *in NUD patients (Figure 5).

#### 6) Eradication regimens as first line treatment

**Figure 6:**
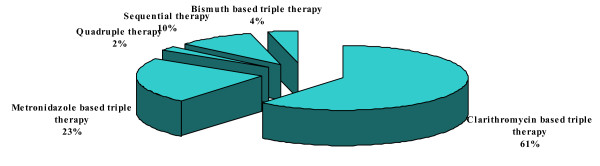
**Eradication regimens as first line treatment**.

The physicians were inquired of their first choice of *H. pylori *eradication regimen. For which various standard regimens were given and they had to select one best first line treatment option. Clarithromycin, amoxicillin, proton pump inhibitor based triple therapy was used as first line treatment by 261 (61%) physicians (Figure 6).

#### 7) Appropriate duration for treating H. pylori infection

Regarding the treatment duration different options were given, 396 (93%) physicians would treat for 7–14 days. Only 30 (7%) physicians wanted to treat for more than 2 weeks.

#### 8) Confirmation of H. pylori eradication

**Figure 7:**
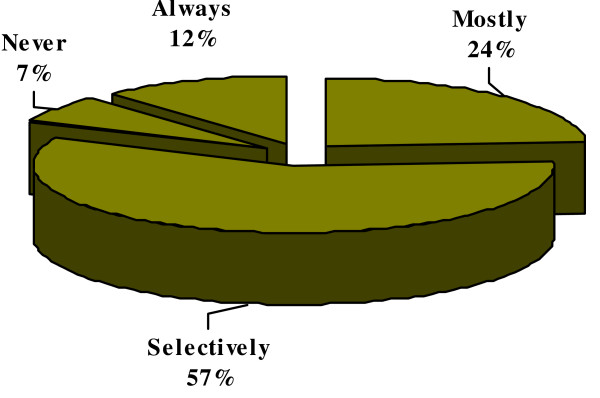
**Confirmation of *H. pylori *eradication**.

Eradication of *H. pylori *was confirmed by 247 (57%) of physicians in selected patients only (Figure 7).

#### 9) Test for documenting H. pylori eradication

*H. pylori *serology was the preferred follow up test for successful eradication by 201 (47%) physicians, followed by 149 (35%) urea breath test. Few physicians 38 (9%) would ask for endoscopy with rapid urease test or endoscopy with histology based testing in 17 (4%). While 21 (5%) physicians would use *H. pylori *stool antigen test for the confirmation of eradication.

#### 10) Treatment plan after failure to eradicate H. pylori infection

Only 155 (36%) of the physicians would refer the patient to gastroenterologist in case of primary treatment failure, the responses to different options are shown in Table 1.

**Table 1:** Treatment plan after failure to eradicate *H. pylori*

Options	Number of doctors	Percentage(%)
Repeat triple therapy	66	15%
Triple therapy with change of antibiotics	86	21%
Quadruple therapy	85	20%
Observation without treatment	34	8%
Refer to gastroenterologist	155	36%

#### 11) Most common source of information about H. pylori infection

Only 135 (32%) primary care physicians were using medical journals as a source of information on *H. pylori *infection followed by text book reading in 101 (23%), pharmaceutical companies provided literature in 77 (18%), respectively. Only 60 (14%) physicians would attend workshop/conferences on any topic to update their knowledge and 53 (13%) physicians would use internet.

## Discussion

This study was aimed to gauge the current practices, understanding and approaches of primary care physicians regarding the management of *H. pylori *infection in the largest city of Pakistan. In the current era the knowledge and evidence in the management of various diseases is expanding very fast and different national and international societies are coming up with evidence based guidelines but their impact at the primary care level is not well studied especially in developing countries. A Korean study revealed that guidelines on the management of *H. pylori *have a little impact on clinical practice in Korea [16]. Another survey from Turkey on 109 general practitioners also pointed out the gaps in practice [17]. Similarly, a study from Italy at 100 randomly selected general practitioners showed that uncertainty seems to persist regarding indications for *H. pylori *treatment, the use of diagnostic testing, and patients follow-up [18].

Our study reflected the existing knowledge of *H. pylori *infection among the primary health care physicians in Karachi, Pakistan. Majority (72%) of physicians in this study believed that *H. pylori *infection is transmitted through contaminated water; however, 14% physicians thought that it was transmitted either through the blood products or by needle stick injuries. Primary care physicians would usually advise *H. pylori *testing in cases of gastric ulcer, duodenal ulcer and dyspepsia in 56%, 46% and 67% of the times respectively. In our study only 19% physicians thought that MALToma is an indication for investigating *H. pylori *and 35% were of the opinion that it should be investigated after surgery for early gastric cancer. Almost similar inferences were concluded from Korean study; in that study 26.9% of primary care physicians would opt for testing *H. pylori *in patients after surgery for early gastric cancer and 13% of the physicians would check it in MALToma [16]. Majority of physicians in the current study preferred serological or biopsy based testing of *H. pylori *in 43% and 32%, respectively. While only 22% of Korean primary care physician opted for serology as a first test for detecting *H. pylori *while 55% of Italian physicians preferred gastroscopy with biopsies as initial diagnostic testing [16,18]. About 88% primary care physicians in this study were treating *H. pylori *for 7–14 days which was similar to Korean study, 90% of Korean physicians were in favor of 7 to 14 days treatment [16]. In this study, only 12% of primary care physicians always ordered follow-up testing after *H. pylori *treatment as compared to Korean primary care physicians who would do follow-up testing in 9.3% cases which is in contrast to Italian physicians' practices. About 50% of Italian physicians thought it was useful to repeat endoscopy with biopsy after treatment to document eradication while 47% of our primary care physicians used serology as follow up test [18]. In contrast the only 6.5% Korean physicians used serology as a follow up test [16]. Only 4.5% physicians in our study prescribed the same regimen after first line treatment failure to eradicate *H. pylori *as compared to 40.7% Korean Physicians who wanted to retreat with same regimen. However, 36% physicians in the current study would refer the patients to gastroenterologist after first treatment failure while only 5.6% of Korean Physicians would do the same. Similarly 25% of Italian physicians were referring to specialist [18]. More than half (55%) of physicians in this study opted to treat *H. pylori *in non ulcer dyspepsia patients as compared to 58.3% of Korean physicians [16]. Medical journals were the source of information/knowledge for 32% of our primary care physicians, while in Turkey the main source of information to primary care physicians was pharmaceutical company sponsored symposia [17].

One of the limitations to this study is that it only reflects the approach of the primary care physicians living in Karachi, Pakistan, which may not be the true reflection of the whole county and also of other parts of the world. However, this is a first study of 426 primary care physicians from South Asia which reflects the existing knowledge, attitude and practices on the management of *H. pylori *in this endemic area. So the findings in our study endorse and highlight the gaps in practices of primary care physicians and the recommended practice guidelines for the management of *H. pylori *infection. The results of this study are pretty much similar to published data from other parts of the world.

## Conclusion

The primary care physicians in this part of the world lack in knowledge regarding management of *H. pylori *infection. Internationally published guidelines and World gastroenterology organization (WGO) practice guideline on *H. pylori *infection for developing countries have little impact on current practices of primary care physicians. We recommend more teaching programs and continuous medical education activities for primary care physicians by government, public and private sector academic institutions.

## Competing interests

The authors declare that they have no competing interests.

## Authors' contributions

SA and MS conceived, designed and coordinated the study. WJ, HAS, SH participated in its design, coordination and manuscript. All authors have read and approved the final manuscript.

## Pre-publication history

The pre-publication history for this paper can be accessed here:

http://www.biomedcentral.com/1471-230X/9/23/prepub

## Supplementary Material

Additional file 1 **Helicobacter pylori infection: Approach of primary care physicians in a developing country**. Performa used in the study. Click here for file
