# Supplementary material for: Helicobacter pylori infection: approach of primary care physicians in a developing country
Source: BMC Gastroenterol. 2009 Apr 9;9:23. doi: 10.1186/1471-230X-9-23 (PMC2678140; doi:10.1186/1471-230X-9-23)
Supplement: Additional file 1 — Helicobacter pylori infection: Approach of primary care physicians in a developing country. Performa used in the study. [file 1471-230X-9-23-S1.doc]

**Questionnaire**

***Helicobacter pylori* infection: Approach of primary care physicians in a developing country**

**Demographic information**

**1) Age in years** ( )

**2) Gender**

Male ( ), Female ( )

**3) Number of years of practicing** ( )

**4) Route of transmission for H. *pylori* infection? (More than one can be true)** 4. a) Contaminated water yes ( ) No ( )

4. b) Iatrogenic spread via Contaminated endoscopes yes ( ) No ( )

4.c ) Through blood products yes ( ) No ( )

4.d ) Through needle injuries yes ( ) No ( )

**5) What are the indications for testing *H. pylori*? (More than one can be true)**

5. a) Dyspepsia yes ( ) No ( )

5. b) Gastric MALToma yes ( ) No ( )

5. c) Gastro esophageal reflex disease yes ( ) No ( )

5. d) Family history of gastric cancer yes ( ) No ( )

5. e) On patient’s request yes ( ) No ( )

5. f) Gastritis yes ( ) No ( )

5. g ) Duodenal Ulcer yes ( ) No ( )

5. h) Gastric Ulcers yes ( ) No ( )

5. i) Patients on long term Proton Pump Inhibitor yes ( ) No ( )

5. J) idiopathic thrombocytopenic purpura yes ( ) No ( )

5. k) Unexplained iron deficiency anemia yes ( ) No ( )

**6) What is the most appropriate test for detection of active *H. Pylori* infection? (Select only one**

**Option)**

6. a) Endoscopy with rapid urease testing yes ( ) No ( )

6. b) Endoscopic biopsy for histology yes ( ) No ( )

6. c) Urea breath test yes ( ) No ( )

6. d) Serology ( Antibodies) yes ( ) No ( )

6. e) Stool antigen test yes ( ) No ( )

**7). Treatment indications for *H. pylori* infection? (More than one can be true)**

7.a) Dyspepsia yes ( ) No ( )

7.b) Duodenal ulcer yes ( ) No ( )

7.c) Gastric ulcer yes ( ) No ( )

7.d) Complicated peptic ulcer disease yes ( ) No ( )

7.e) MALToma yes ( ) No ( )

7.f) Atrophic gastritis yes ( ) No ( )

7.g) Post gastric cancer resection yes ( ) No ( )

7.h) Unexplained iron deficiency anemia yes ( ) No ( )

7.i) Idiopathic thrombocytopenic purpura yes ( ) No ( )

**8). Which eradication regimen will you chose as first line treatment? (Select only one option)**

8.a) Proton pump inhibitors + Clarithromycin + Amoxicillin yes ( ) No ( )

8.b) Proton pump inhibitors + Clarithromycin + Metronidazole yes ( ) No ( )

8.c) Bismuth citrate + Clarithromycin +Furazolidone yes ( ) No ( )

8.d) Furazolidone+Amoxicillin+PPI+ Bismuth citrate yes ( ) No ( )

8.e) Sequential therapy (5 days PPI+ Amoxicillin

followed by 5 days PPI+ Clarithromycin+ Tinidazole) yes ( ) No ( )

**9) What is the appropriate duration for treating *H. pylori* infection? (Select only one option)**

9.a) One week yes ( ) No ( )

9.b) 10 days yes ( ) No ( )

9.c) two weeks yes ( ) No ( )

9.d) more than 2 weeks yes ( ) No ( )

**10) Will you confirm *H. pylori* eradication? (Select only one option)**

11.a) Mostly yes ( ) No ( )

11.b) Selectively yes ( ) No ( )

11.c) Never yes ( ) No ( )

11.d) Always yes ( ) No ( )

**11) What test will you order to document *H. pylori* eradication? (Select only one option)**

12.a) Endoscopy with rapid urease testing yes ( ) No ( )

12.b)Endoscopic biopsy for histology yes ( ) No ( )

12.c) Urea breath test yes ( ) No ( )

12.d) Serology ( Antibodies) yes ( ) No ( )

12.e) Stool antigen test yes ( ) No ( )

**12) Treatment plan after failure to eradicate *H pylori*? (Select only one option)**

13.a) Repeat of triple therapy yes ( ) No ( )

13.b) Triple therapy with change of antibiotics yes ( ) No ( )

13.c) Quadruple therapy yes ( ) No ( )

13.d) Observation without treatment yes ( ) No ( )

13.e) Refer to gastroenterologist yes ( ) No ( )

**13) Do you prescribe *H. pylori* eradication in non ulcer dyspepsia (NUD) ? (Select only one option)**

14.a) Always yes ( ) No ( )

14.b) Sometimes yes ( ) No ( )

14.c) When requested yes ( ) No ( )

14.d) Never yes ( ) No ( )

**14) What is the most common sources of information about *H. Pylori*? (Select only one option)**

16.a) Medical journals yes ( ) No ( )

16.b) Pharmaceutical company-sponsored symposia yes ( ) No ( )

16.c) Textbooks yes ( ) No ( )

16.d) Conferences yes ( ) No ( )

16.e) On-line sites yes ( ) No ( )
